# Composite Fiber Wrapping Techniques for Enhanced Concrete Mechanics

**DOI:** 10.3390/polym16192820

**Published:** 2024-10-05

**Authors:** Zhongxu Li, Guojun Hao, Haoran Du, Tianjian Fu, Depei Liu, Yuxiang Huang, Yongcheng Ji

**Affiliations:** 1 College of Aulin, Northeast Forestry University, Harbin 150040, China; zhongxuli@nefu.edu.cn (Z.L.); haoguojun123@126.com (G.H.); duhaoranharbin@163.com (H.D.); zane@nefu.edu.cn (T.F.); liudepei0531@163.com (D.L.); rickesthyx@nefu.edu.cn (Y.H.); 2 College of Civil Engineering and Transportation, Northeast Forestry University, Harbin 150040, China

**Keywords:** fiber-reinforced polymer (FRP), single-layer reinforcement, double-layer reinforcement, composite reinforcement, ABAQUS finite element simulation

## Abstract

This study systematically investigates the enhancement effects of different fiber-reinforced polymer (FRP) materials on the axial compressive performance of concrete. Through experimental evaluations of single-layer, double-layer, and composite FRP reinforcement techniques, the impact of various FRP materials and their combinations on concrete’s axial compressive strength and deformation characteristics was assessed. The results indicate that single-layer CFRP reinforcement significantly improves concrete axial compressive strength and stiffness, while double-layer CFRP further optimizes stress distribution and load-bearing capacity. Among the composite FRP reinforcements, the combination with CFRP as the outer layer demonstrated superior performance in enhancing the overall structural integrity. Additionally, numerical analyses of the mechanical behavior of the reinforced structures were conducted using ABAQUS 2023HF2 finite element software, which validated the experimental findings and elucidated the mechanisms by which FRP influences the internal stress field of concrete. This research provides theoretical support and empirical data for the optimized design and practical application of FRP reinforcement technologies in engineering.

## 1. Introduction

**Figure 1:**
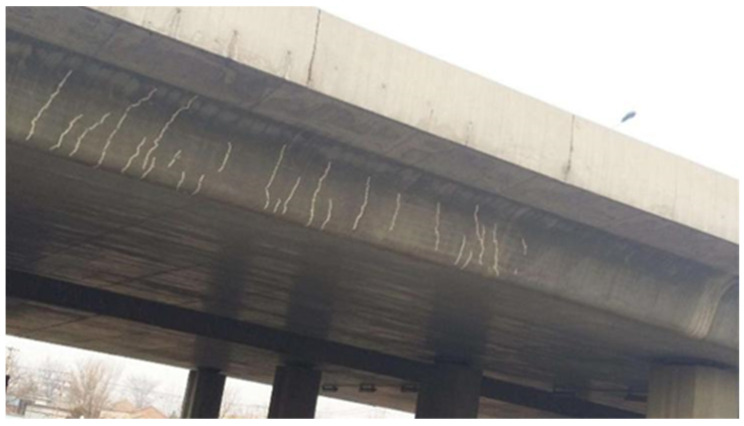
Cracks in concrete bridge.

As the scale and complexity of modern construction continue to grow, concrete structures face many challenges during their long-term service life. Concrete structures are susceptible to chemical corrosion, physical abrasion, and fatigue damage, particularly in harsh environmental conditions, such as seismic zones or regions with extreme climates. These factors can lead to the propagation of cracks, degradation of strength, and reduced stiffness. Figure 1 illustrates the development of cracks in a concrete bridge over its service life. These cracks compromise the structural integrity and pose significant economic and social risks.

Traditional concrete reinforcement techniques, such as concrete jacketing, steel plate reinforcement, and section enlargement, can enhance the load-bearing capacity of structures. However, these methods have notable drawbacks, including complex construction processes, high material consumption, and significant increases in structural weight. For instance, concrete jacketing involves wrapping the existing structure with a new layer of concrete to improve its load capacity and durability. This approach, however, is time-consuming, and the additional concrete layer increases the overall size and weight of the structure, imposing extra burdens on the existing foundation, especially when the foundation’s load-bearing capacity is limited [1]. Steel plate reinforcement can effectively enhance tensile and shear capacities but has significant limitations. First, steel plates are highly susceptible to corrosion in humid or chemically aggressive environments, requiring additional protective measures and increasing construction and maintenance costs [2]. Second, installing steel plates involves extensive welding, making the process complex and requiring a high level of craftsmanship [3]. The section enlargement method, while applicable to various types of structures, is also complex and significantly impacts the dimensions of the existing structure. This technique involves using a large amount of concrete, notably increasing the structure’s weight [4]. Additionally, section enlargement requires temporary supports and a prolonged curing period, imposing stringent demands on construction conditions and project timelines. Therefore, developing a new structural reinforcement technology that is efficient, lightweight, and corrosion-resistant has become a pressing challenge in engineering.

Fiber-reinforced polymer (FRP) has become a focal point in research on concrete structure reinforcement technologies due to its high strength, low density, and excellent corrosion resistance. Encasing concrete structures with FRP can effectively enhance compressive strength, stiffness, and ductility, thereby extending the service life of the structures. Moreover, compared with traditional reinforcement methods, FRP offers significant advantages regarding ease of construction and long-term effectiveness. Dai et al. [5] demonstrated that the ultimate load-bearing capacity of concrete is significantly improved when using high-deformation FRP, particularly in enhancing structural ductility and seismic performance. Additionally, FRP effectively mitigates stress concentration issues commonly associated with traditional reinforcement methods, further prolonging the service life of concrete structures. Liu et al. [6] conducted an in-depth study on the mechanical properties of lightweight aggregate concrete reinforced with carbon fiber-reinforced polymer (CFRP). Their findings revealed that CFRP reinforcement could substantially enhance the mechanical properties of the specimens, increasing compressive strength by up to 1.5 times the original strength. This study confirmed the significant impact of CFRP in enhancing concrete’s mechanical performance. It developed a mathematical model relating peak stress to corresponding strain, providing crucial theoretical support for reinforcement design. Ola et al. [7] investigated the performance of CFRP-reinforced recycled aggregate concrete beams, monitoring key performance parameters such as initial cracking load, crack patterns, and ultimate deflection. The results indicated that CFRP-reinforced beams exhibited significantly higher ultimate load-bearing capacity than their unreinforced counterparts. Additionally, CFRP reinforcement led to noticeably lower deflection at maximum load, indicating that CFRP not only effectively enhanced load-bearing capacity but also improved the deformation performance of beams. Ouyang et al. [8] conducted comparative seismic performance tests on reinforced concrete square columns constrained by basalt fiber-reinforced polymer (BFRP) and CFRP. The study found that BFRP- and CFRP-reinforced columns have similar load-bearing capacities, but BFRP-reinforced columns exhibited superior ductility and energy dissipation capacity, demonstrating better seismic performance. Furthermore, both types of FRP reinforcement significantly improved the shear capacity of the reinforced concrete columns, shifting the failure mode from shear–flexural failure to ductile flexural failure. Ahmed et al. [9] performed tests on FRP-reinforced T-beams, analyzing the effects of FRP quantity, vertical and horizontal bonding angles, and end anchorage methods on reinforcement effectiveness. The results showed that single-strip reinforcement methods were less beneficial for enhancing overall load-bearing capacity. Therefore, careful consideration should be given to single-strip reinforcement methods in design to avoid adverse effects on the overall structural performance due to local failures. Raza et al. [10] studied concrete columns reinforced with different types of FRP. The results indicated that FRP effectively constrained concrete members and inhibited microcrack development, thereby enhancing compressive load-bearing capacity and deformation ability. The study further pointed out that, excluding the effects of initial concrete strength and member geometry, the strength and thickness of the FRP were key factors influencing the final load-bearing capacity of the structure.

While many researchers have demonstrated the effectiveness of FRP reinforcement technology in enhancing structural load-bearing capacity, most studies have focused on the effects of a single type of FRP reinforcement. This research, however, is comprehensive in its approach, exploring the impact of composite reinforcement using different FRP combinations on structural performance. It specifically focuses on the synergistic effects of varying numbers of wrapping layers and FRP-type combinations under external loading conditions. The study investigates the effects of single-layer, double-layer, and composite double-layer FRP on the compressive performance of concrete, with a keen eye on the synergistic effects of different FRP combinations under load. The use of finite element analysis (FEA) to simulate FRP-reinforced structures, validate experimental results, and gain deeper insights into the reinforcement mechanisms further adds to the comprehensive nature of this research. The ultimate goal is to fill the gaps in the study of multi-layer FRP combinations, mechanical behavior, and reinforcement mechanisms, providing more comprehensive and in-depth theoretical support and empirical evidence for the application of this technology, thereby promoting its broader adoption in the field of concrete structure reinforcement.

## 2. Material and Method

### 2.1. Materials

#### 2.1.1. Fiber-Reinforced Polymer (FRP)

**Figure 2:**
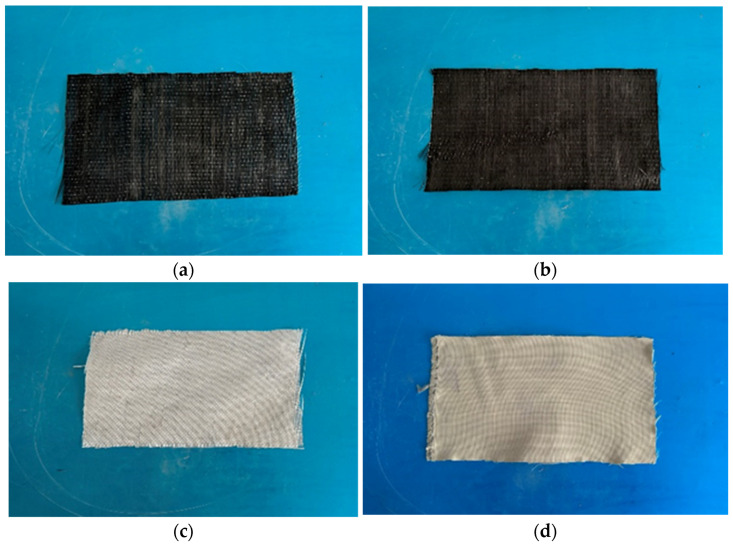
Different types of FRP: (**a**) CFRP; (**b**) BFRP; (**c**) GFRP; (**d**) AFRP.

This study used four types of fiber-reinforced polymers (FRP) to reinforce concrete specimens. These FRPs, as shown in Figure 2, include carbon fiber-reinforced polymer (CFRP), basalt fiber-reinforced polymer (BFRP), glass fiber-reinforced polymer (GFRP), and aramid fiber-reinforced polymer (AFRP). The FRPs were sourced from Toray Industries, Inc. (Tokyo, Japan), Anjie Composite Materials Co., Ltd. (Haining, China), Qingcheng Construction Technology Co., Ltd. (Chiayi City, China), and East China Materials Research Institute, respectively. Each of these materials possesses high tensile strength and elastic modulus, making them highly effective in reinforcing concrete structures. Based on the relevant test data provided by the manufacturer, Table 1 presents the fundamental physical properties of the four types of FRP used in this study, which comply with the requirements of GB 50728-2011 [11].

Epoxy resin is commonly used as an adhesive for bonding FRP to structures due to its excellent bonding strength at room temperature and ease of application. However, recent studies have shown that using inorganic matrix adhesives as the bonding material for FRP can significantly improve the durability of the FRP–concrete interface under high-temperature conditions [12]. While inorganic matrix adhesives demonstrate superior durability in extreme conditions such as high temperatures, considering the lower cost and widespread application of epoxy resin in practical engineering, it remains a valuable reference material for bonding. Therefore, this study utilizes epoxy resin as the adhesive for bonding FRP to concrete, aiming to evaluate its reinforcement performance under conventional environmental conditions.

**Figure 3:**
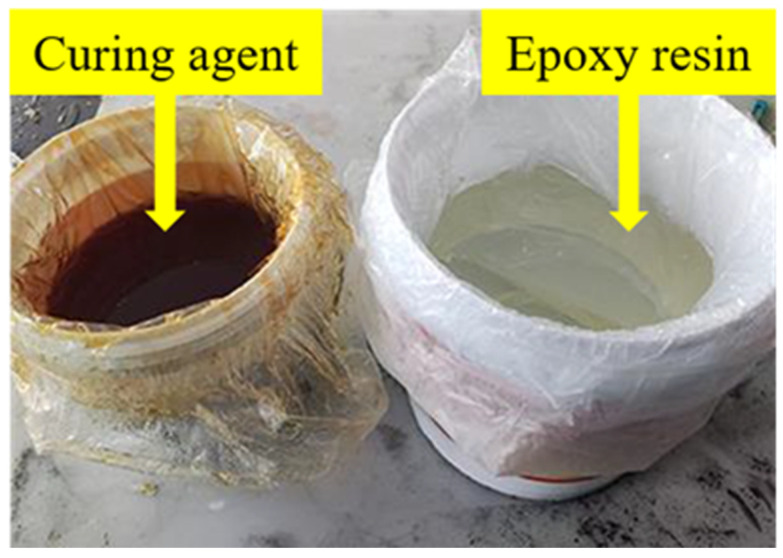
Adhesive resin glue.

A specially formulated resin adhesive was selected for use in this study to ensure adequate bonding of the FRP to the concrete surface. The adhesive consists of two components, A and B, as shown in Figure 3. Component A is an epoxy resin, a translucent milky white substance known for its excellent adhesion and chemical resistance. Component B is a hardener characterized by a dark red color, which, when mixed with component A, facilitates the curing reaction of the resin. The epoxy resin and hardener were thoroughly mixed in a 2:1 ratio to ensure the formation of a robust bond layer between the FRP and the concrete surface, thereby enhancing the reinforcement effectiveness.

#### 2.1.2. Aggregate

**Figure 4:**
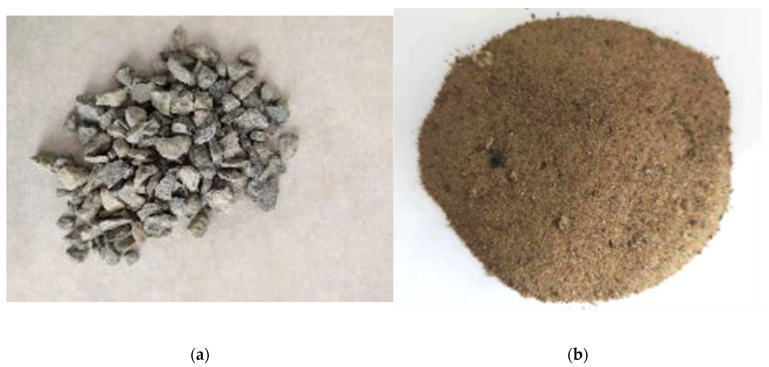
Aggregates: (**a**) coarse aggregate; (**b**) fine aggregate.

**Figure 5:**
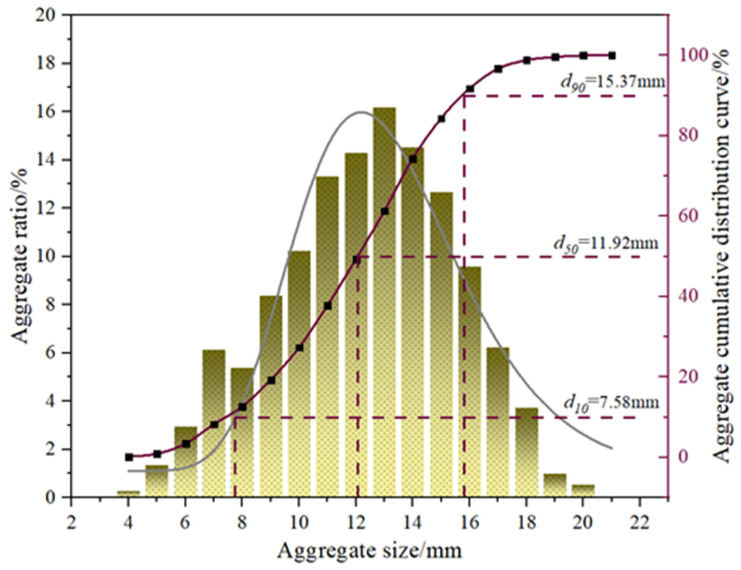
Coarse aggregate particle size distribution.

The coarse aggregate used in the experiment was limestone, as shown in Figure 4a. According to GB/T 14685-2011 [13], the coarse aggregate was classified into two size ranges, 5–10 mm and 10–20 mm, and mixed in a 3:7 ratio. Figure 5 illustrates the particle size distribution of the coarse aggregate within the concrete specimens. Figure 4b shows the fine aggregate of natural river sand. The fine aggregate’s water content, mud content, and fineness modulus were 0.33%, 2.8%, and 2.1, respectively, meeting the requirements of JGJ52-2006 [14].

#### 2.1.3. Cement

The cement used in this study was P.O 42.5 ordinary Portland cement produced by Hongtai Group. The fineness, mechanical properties, and other relevant indicators of the cement are presented in Table 2, all of which meet the requirements of GB 175-2007 [15].

#### 2.1.4. Water

The water used in the experiment was tap water from Harbin, with all parameters meeting the requirements of JGJ 63-2006 [16].

### 2.2. Specimen Preparation and Test Methods

#### 2.2.1. Specimen Mix Proportion

**Figure 6:**
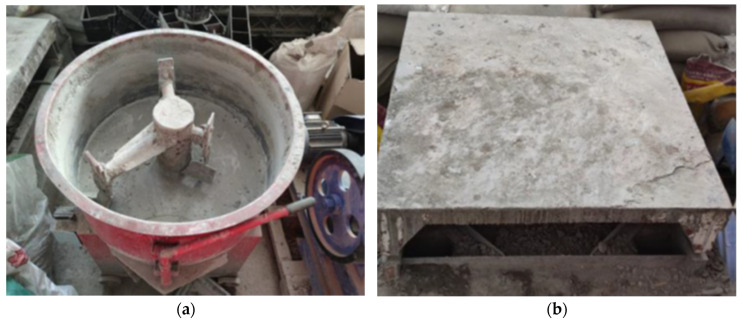
Equipment used for specimen preparation: (**a**) concrete mixer; (**b**) mechanical vibration platform.

All specimens were standardized as cylindrical shapes with a diameter of 100 mm and a height of 200 mm to prevent stress concentration during FRP reinforcement. Considering that C30 concrete is widely used in practical engineering applications, the concrete strength grade for the experiment was selected as C30. The specific mix proportions are shown in Table 3. According to GB/T 50080-2002 [17], the coarse and fine aggregates and the cement were first mixed using a mixer. Water was then gradually added while mixing continued. The concrete mixture was poured into molds and vibrated to ensure uniform compaction, with the surface slurry removed. Figure 6 shows the mixer and vibration platform used in the experiment. The molds were removed 24 h after casting, and the specimens were cured under standard conditions for 28 days.

#### 2.2.2. FRP Bonding

After the curing process, the specimens were wrapped with FRP according to the design scheme listed in Table 4. The study employed a full factorial design to ensure a comprehensive examination of the reinforcement effects of different FRP combinations. This approach provides a thorough representation of the impact of various FRP types and their combinations on the performance of concrete structures. By systematically testing all experimental combinations, the study gains deeper insights into the synergistic effects between different FRP materials, offering more comprehensive data support for further theoretical analysis and model validation.

The FRP was adhered to using the previously described epoxy resin adhesive. First, the surface of the specimens was sanded and cleaned to ensure that no loose particles or dust remained on the bonding surface. Next, the epoxy resin adhesive, mixed in the specified ratio, was evenly applied to the specimen surface. Finally, the pre-cut FRP sheets were carefully applied to the specimen surface, ensuring no air bubbles or wrinkles. For specimens requiring double-layer or composite wrapping, the second layer was applied after the first layer of FRP had fully cured. Considering the potential synergistic effects between different types of FRP, the study also included composite wrapping methods involving different FRP types, in addition to the double-layer wrapping of the same FRP. This approach aims to maximize the advantages of each FRP type and enhance the overall performance of the concrete structure through their combined synergistic effects.

### 2.3. Axial Compression Test

**Figure 7:**
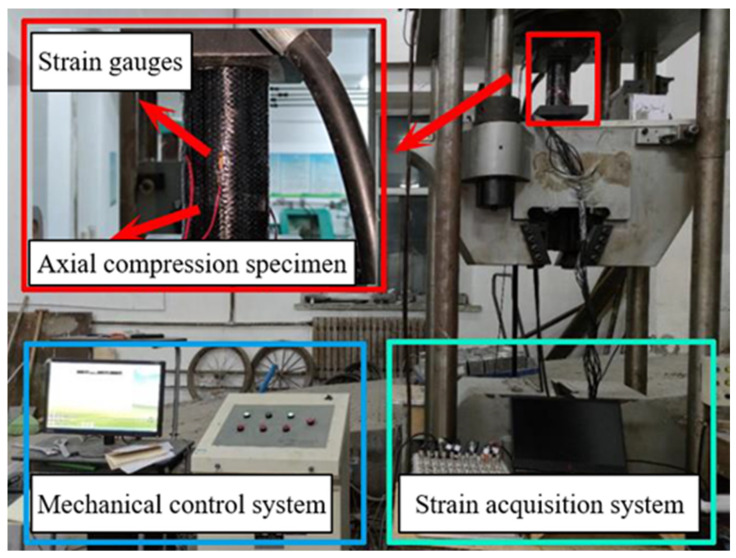
Hydraulic servo.

The uniaxial compression test of the concrete specimens was conducted by GB/T 50081-2019 [18]. The servo-hydraulic testing machine used for the experiment was a YAM-5000F model with a maximum capacity of 2000 kN. As shown in Figure 7, the servo-hydraulic machine consists of a mechanical control system and a data acquisition system, which control the load magnitude, loading speed, and data collection. Each group of specimens underwent three tests, and the axial compressive strength was calculated according to Equation (1). The final result for each group was calculated as the average of the three tests. The median value was selected if the error between the maximum or minimum value and the median value exceeded 15%. Additionally, to measure the axial strain of the specimens under axial compression, vertical strain gauges were attached at the mid-height of the specimens. The data collection was carried out using an ASMB2-16 static strain acquisition system.

(1) $$f_{cc}=\frac{4F}{\pi d^{2}}$$

## 3. Results and Discussion

FRP material and wrapping method significantly influence concrete’s axial compressive strength [19]. The axial compressive strength, ultimate compressive strain, and elastic modulus of each group of specimens were tested, and the results are presented in Table 5. The elastic modulus was determined as the secant modulus, calculated as the slope of the line corresponding to 40% of the ultimate compressive stress on the ascending portion of the stress–strain curve [20].

### 3.1. Single-Layer FRP Reinforcement

#### 3.1.1. Axial Compressive Strength

**Figure 8:**
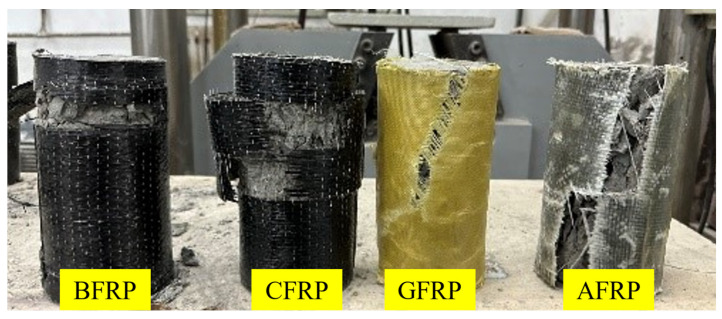
Axial compression failure of specimens reinforced with single-layer FRP.

Figure 8 illustrates the failure modes of concrete specimens reinforced with different single-layer FRP under axial compression testing. The observed variations in the failure patterns of the four FRP-reinforced specimens indicate significant differences in the mechanisms and effectiveness of each FRP type in enhancing the axial compressive strength of concrete [19]. In the NC specimen, partial debonding of the CFRP was noted during compression, but overall, it continued to provide effective confinement to the concrete. The failure predominantly occurred within the concrete, accompanied by minor external crack propagation during the axial compression. The NB specimen exhibited a failure mode similar to that of NC, with more pronounced fiber tearing and a greater extent of FRP debonding under axial load. Nonetheless, the BFRP effectively delayed the failure of the concrete, and its wrapping layer remained largely intact, indicating that BFRP provides good confinement while maintaining integrity under high deformation.

In contrast, NG and NA specimens performed relatively weaker in the axial compression tests. As shown in Figure 8, both NG and NA experienced significant fiber cracking and concrete spalling during the tests. The tensile strength and tensile elastic modulus of GFRP and AFRP are comparatively lower, making it difficult for them to provide adequate confinement under high compressive stress. This insufficiency led to rapid crack propagation within the concrete, resulting in structural failure [21,22]. The observed failure modes suggest that while GFRP and AFRP contribute to increased axial compressive strength, their impact on ductility is relatively limited. Particularly under high pressure, these materials struggle to confine the concrete, effectively leading to earlier failure.

**Figure 9:**
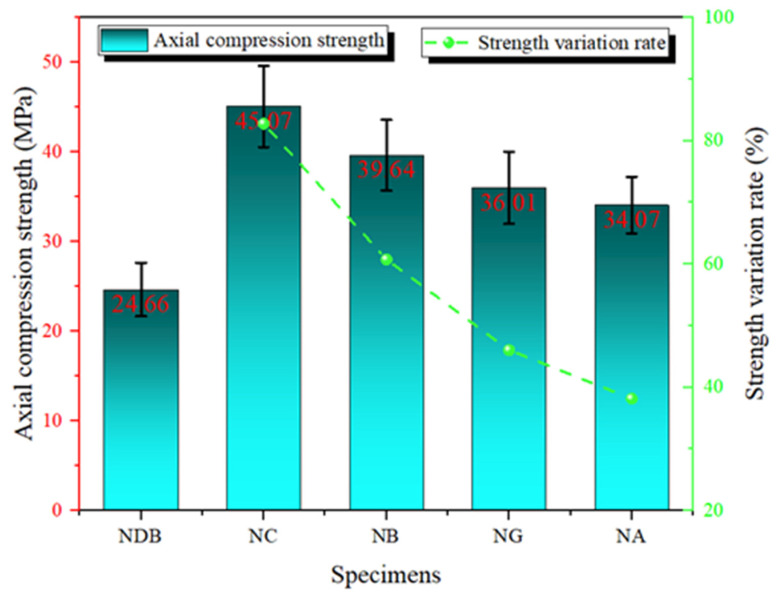
Axial compressive strength of specimens reinforced with single-layer FRP.

The axial compressive strength and the corresponding improvement rates of the concrete specimens reinforced with the four types of FRP are shown in Figure 9. It can be observed that the axial compressive strength of the specimens reinforced with a single layer of FRP increased by approximately 38.2–82.8%. This result is comparable to the findings of Qaidi et al. [23] where the improvement ranged from 58.9% to 87.8%. This indicates that although the specific enhancement values vary across different studies, the overall trend remains consistent. Compared with the NDB specimen, all FRP-reinforced specimens exhibited significant improvements in axial compressive strength. Among the single-layer FRP-reinforced specimens, NC demonstrated the best performance, with an axial compressive strength of 45.07 MPa, representing an 82.77% increase over the NDB specimen. This substantial enhancement is primarily attributed to CFRP’s high tensile strength and elastic modulus, which provide strong confinement during compression, significantly enhancing the concrete’s compressive capacity. The NB specimen showed a 60.75% improvement in axial compressive strength compared with the NDB specimen. Although BFRP has a lower elastic modulus than CFRP, its relatively high strength still offers effective reinforcement for the concrete. The NG specimen achieved an axial compressive strength of 36.01 MPa, with an improvement rate of 46.03%. The reinforcement effect of GFRP was noticeably lower than that of CFRP and BFRP. However, its higher elongation allowed it to absorb more energy during the deformation process, delaying failure and, thus, improving the specimen’s load-bearing capacity [24]. AFRP exhibited the lowest improvement in concrete axial compressive strength, with an enhancement rate of 38.16%. Due to its relatively lower tensile strength, AFRP’s confinement capacity under high pressure was less effective than the other FRPs, resulting in a more limited improvement.

#### 3.1.2. Stress–Strain Curves

**Figure 10:**
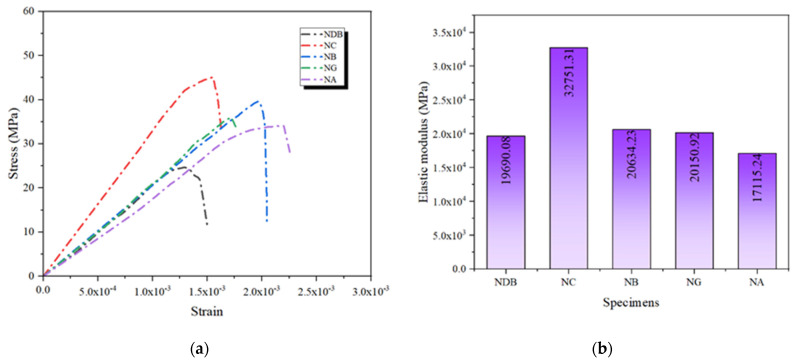
Axial compressive performance of specimens reinforced with single-layer FRP: (**a**) stress–strain curves; (**b**) elastic modulus.

The stress–strain curves demonstrate the effectiveness of FRP in enhancing the specimens’ strength and reveal their differences in accommodating deformation. Figure 10a shows the stress–strain curves of concrete specimens reinforced with different single-layer FRPs. From the shape of the curves and the variation in peak stress, the distinctions among the four types of FRP in improving the axial compressive performance of concrete are evident. The NC specimen exhibits the highest peak stress and the steepest ascending segment on the stress–strain curve, indicating that CFRP can rapidly enhance the load-bearing capacity of the specimen under axial compression while imparting a high degree of stiffness. This performance is closely related to CFRP’s high tensile strength and elastic modulus. These characteristics enable CFRP to provide effective confinement to the concrete during the initial loading phase, thereby quickly increasing the specimen’s load-bearing capacity. Compared to NC, the peak stress of NB is slightly lower, but it still outperforms the other FRP-reinforced specimens. The curve for NB is relatively gentler, indicating that its reinforcement effect balances strength and ductility, allowing the specimen to sustain more significant deformation while maintaining a high load-bearing capacity [25]. This demonstrates that BFRP provides sufficient stiffness and effectively enhances the ductility of the concrete.

The stress–strain curve for NG exhibits significant ductility but relatively low peak stress. This is due to the lower elastic modulus of GFRP, which limits its ability to constrain the concrete during loading, causing the specimen to fail at lower stress levels. As a result, while GFRP can maintain a specific load-bearing capacity over a more extensive strain range, its reinforcement effect under high-stress conditions is inferior to that of CFRP and BFRP. Among the four single-layer FRP-reinforced specimens, the NA specimen’s stress–strain curve shows the earliest decline in stress, indicating that it reached its limit at relatively lower stress levels. This is primarily due to the lower tensile strength and elastic modulus of AFRP, which results in poorer reinforcement performance under high stress, leading to early failure [26].

#### 3.1.3. Elastic Modulus

A comparative analysis of the elastic modulus for each group was conducted to assess the effectiveness of different FRP materials in enhancing the stiffness of the specimens, as shown in Figure 10b. The NC specimen exhibited a significantly higher elastic modulus than the other FRP-reinforced groups, reaching 3.275 × 10^4^ MPa. This indicates that CFRP provides substantial confinement to the concrete during the initial loading phase, allowing it to withstand more significant stress with minimal deformation. The elastic moduli of NB and NG were similar, at 2.063 × 10^4^ and 2.015 × 10^4^ MPa, respectively, and were lower than that of NC. However, their moderate stiffness allows for a balance between strength and deformation capacity. The NA specimen had the lowest elastic modulus, at just 1.712 × 10^4^ MPa, further confirming that AFRP is less effective in enhancing the specimen’s stiffness under high compressive conditions. These results indicate a significant difference in the ability of various FRP materials to improve the overall stiffness of concrete structures, with CFRP being the most effective. At the same time, AFRP is relatively less [27].

### 3.2. Double-Layer FRP Reinforcement

#### 3.2.1. Compressive Strength

**Figure 11:**
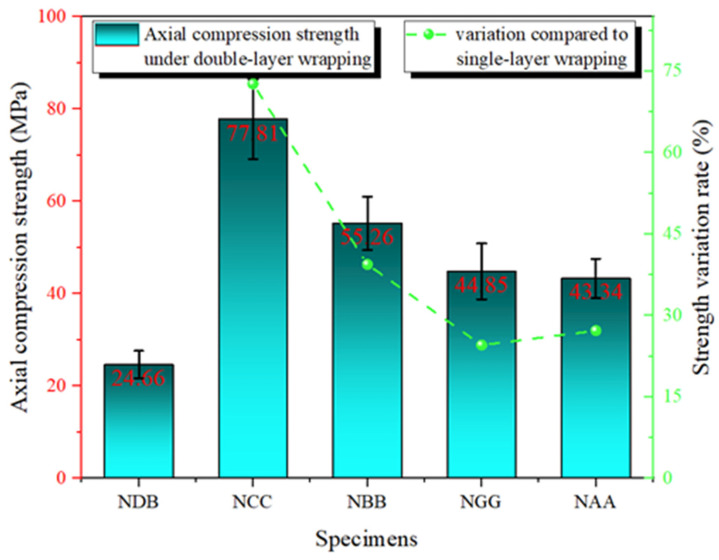
Axial compressive strength of specimens reinforced with double-layer FRP.

Under double-layer FRP reinforcement, the axial compressive strength of concrete specimens showed a significant improvement compared to those reinforced with single-layer FRP. Figure 11 presents the axial compressive strength and the rate of change for the double-layer FRP-reinforced specimens. The results indicate that the NCC specimen achieved an axial compressive strength of 77.81 MPa, approximately three times that of the NDB specimen. This substantial increase can be attributed to three key factors: (1) The double-layer CFRP provides greater stiffness and stronger confinement, significantly enhancing the concrete’s ability to resist crack propagation under compressive loading [28]. (2) The double-layer FRP wrapping results in a more uniform stress distribution, alleviating local stress concentration issues observed in single-layer FRP and further enhancing the overall structural strength. (3) The epoxy resin adhesive is also crucial in double-layer reinforcement. The improved bonding performance strengthens the interface between the FRP layers and between the FRP and concrete, preventing early interface delamination and enhancing the overall mechanical performance [29].

NBB exhibited an axial compressive strength of 55.26 MPa under double-layer reinforcement, significantly improving compared to the single-layer BFRP reinforcement. The double-layer BFRP increased the effective confinement area, enhancing the sealing effect on the concrete and improving its axial compressive performance. The axial compressive strengths of NGG and NAA also increased to 44.85 and 43.34 MPa, respectively, demonstrating the potential of double-layer FRP reinforcement technology to enhance axial compressive strength further. However, due to the inherently lower elastic moduli of GFRP and AFRP, their effectiveness in improving structural strength is relatively limited.

#### 3.2.2. Stress–Strain Curves

**Figure 12:**
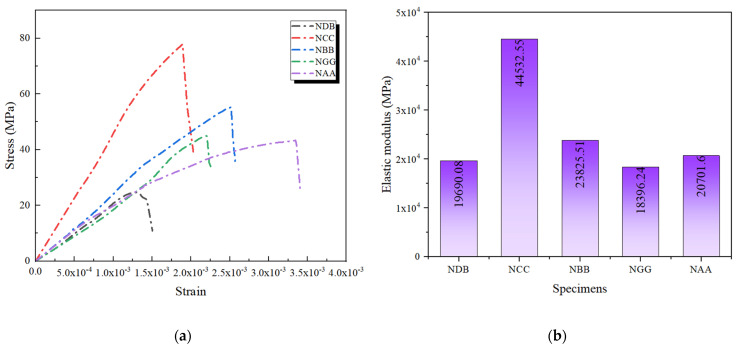
Axial compressive performance of specimens reinforced with double-layer FRP: (**a**) stress–strain curves; (**b**) elastic modulus.

Figure 12a presents the stress–strain curves of the unreinforced specimens and those reinforced with double-layer FRP, providing further insights into the characteristics of double-layer FRP reinforcement. The double-layer FRP specimens exhibited noticeable improvements in peak stress and strain compared with single-layer reinforcement. The double-layer CFRP-reinforced specimens demonstrated greater strain capacity before reaching ultimate strength. This phenomenon indicates that double-layer FRP reinforcement provides high strength and enhances the structure’s ductility [30]. For the specimens reinforced with double-layer FRP, the experimental results showed an increase in axial compressive strength ranging from approximately 75.8% to 215.5%. In comparison, the findings by Ma et al. [31] and Ranolia et al. [32] indicated an improvement of around 26% to 142%. This discrepancy can be attributed to the fact that the concrete used in our study was designed to a C30 strength grade as FRP tends to have a more pronounced strengthening effect on lower-grade concrete. The stress–strain curve of NBB was gentler than that of the single-layer BFRP, indicating that it could maintain a high load-bearing capacity even under more significant deformation. This can be attributed to the more uniform stress distribution and higher resistance to deformation provided by the double-layer BFRP. The stress–strain curves of NGG and NAA also showed some improvement. However, due to the lower elastic moduli of GFRP and AFRP, the stiffness enhancement during loading was not as significant as that observed with CFRP and BFRP. Consequently, the stress–strain curves of NGG and NAA exhibited an earlier decline during the increase in stress, indicating that their ability to support concrete under high-strain conditions is relatively limited.

#### 3.2.3. Elastic Modulus

The impact of double-layer FRP reinforcement on the elastic modulus of concrete specimens is clearly demonstrated in Figure 12b. With double-layer CFRP reinforcement, the elastic modulus of the NCC specimen reached 4.453 × 10^4^ MPa, which was significantly higher than the 3.275 × 10^4^ MPa observed with single-layer wrapping. This indicates that double-layer CFRP enhances the specimen’s ability to resist deformation during the initial phase of axial loading, thereby improving overall stiffness. The NBB specimen also exhibited a notable increase in elastic modulus, reaching 2.383 × 10^4^ MPa, further validating the effectiveness of double-layer FRP reinforcement in enhancing structural stiffness and load-bearing capacity.

Although the elastic modulus of the NAA specimen showed some improvement, it remained significantly lower than that of specimens reinforced with CFRP and BFRP. It suggests that despite the increased thickness from double-layer reinforcement, the inherent low stiffness of AFRP limits its contribution to overall stiffness. However, double-layer reinforcement improved interface performance and stress distribution, improving toughness and ductility under more extensive deformation conditions. A slight reduction in the elastic modulus of the NGG specimen compared with NG was observed, which can be attributed to several factors:

1. In double-layer GFRP reinforcement, an additional interface between the FRP layers and the concrete introduces a new contact surface. If the epoxy resin adhesive is not applied uniformly, it may increase porosity at the interface, thereby reducing overall stiffness.
2. Interlayer slippage can occur in double-layer GFRP reinforcement, weakening stress transfer efficiency, particularly pronounced in materials like GFRP with a lower elastic modulus.
3. The uneven stress distribution is a crucial factor.

Under double-layer GFRP reinforcement, the inner and outer layers experience different stress levels, diminishing the inner layer’s contribution to overall stiffness. Thus, a combination of factors—including bonding technique, interface effects, interlayer slippage, and uneven stress distribution—contributed to the reduction in NGG’s elastic modulus.

### 3.3. Composite FRP Reinforcement

#### 3.3.1. CFRP as the Outer Layer

**Figure 13:**
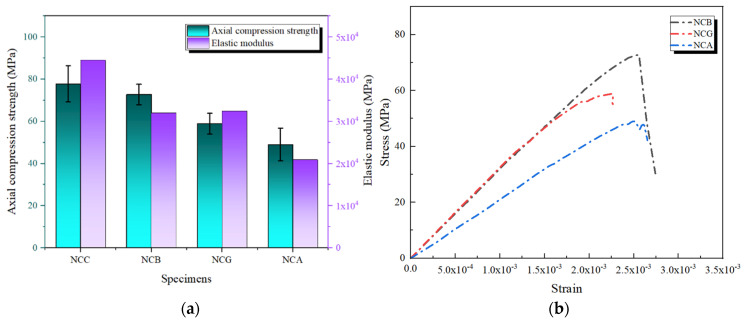
Axial compressive performance of composite reinforcement with CFRP as the outer layer: (**a**) axial compressive strength and elastic modulus; (**b**) stress–strain curves.

Using high-strength, high-modulus CFRP as the outer layer in composite FRP concrete reinforcement can enhance ductility and crack resistance while maintaining high structural stiffness. Figure 13 illustrates the mechanical performance of concrete specimens reinforced with CFRP and other FRP combinations. In composite FRP reinforcement, when CFRP is used as the outer layer, the axial compressive performance of the concrete specimens is significantly improved. This improvement is primarily due to the high tensile strength and elastic modulus of CFRP and its synergistic effect with the inner layers of BFRP, GFRP, and AFRP. As shown in Figure 13 and Table 5, the axial compressive strength of the NCB specimen reached 72.72 MPa, a marked increase compared with single-layer CFRP reinforcement. On the one hand, the outer CFRP provided greater stiffness and higher initial confinement during loading [33]. On the other hand, BFRP, as the inner layer, alleviated stress concentration within the specimen due to its good ductility, further enhancing the overall load-bearing capacity of the specimen.

The stress–strain curves in Figure 13b reveal the reinforcement characteristics when CFRP is used as the outer layer. It can be observed that the stress–strain curves of the composite FRP specimens exhibit a pronounced elastic phase before reaching the ultimate stress. In particular, the NCB specimen shows an apparent linear enhancement in the ascending segment of the stress curve, indicating that the high elastic modulus of CFRP effectively controls the deformation of the concrete during the initial loading phase. BFRP is used as the inner layer, acts as a buffer, and increases the strain capacity, allowing the structure to maintain a high load-bearing capacity over a more extensive deformation range [34]. For NCG and NCA, the elastic moduli reached 3.248 × 10^4^ MPa and 2.090 × 10^4^ MPa, respectively, but their axial compressive strengths were still lower than NCB’s. The lower elastic moduli of GFRP and AFRP limit their ability to provide stiffness comparable to the CFRP outer layer under high pressure, constraining the synergistic effect between the composite layers. Particularly for NCG, the stress–strain curve shows early instability after reaching the peak stress, reflecting the insufficient confinement provided by the inner GFRP layer under high stress, leading to earlier failure of the specimen.

#### 3.3.2. BFRP as the Outer Layer

**Figure 14:**
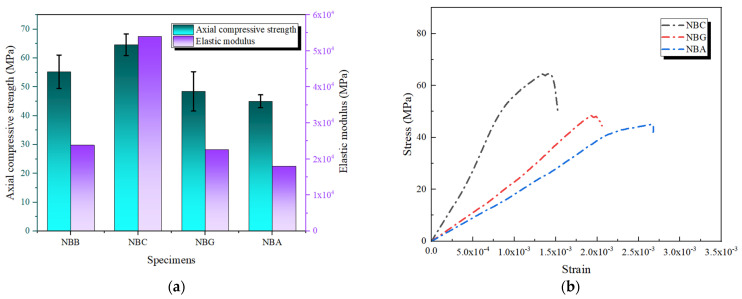
Axial compressive performance of composite reinforcement with BFRP as the outer layer: (**a**) axial compressive strength and elastic modulus; (**b**) stress–strain curves.

The concrete specimens exhibit different mechanical characteristics when BFRP is used as the outer reinforcement layer. Compared with CFRP as the outer layer, BFRP offers more excellent ductility and better fatigue resistance. Consequently, it demonstrates better energy absorption capacity and ductility in axial compression tests. Figure 14 presents the axial compressive performance of the composite FRP-reinforced specimens with BFRP as the outer layer. The axial compressive strength of NBC reached 64.59 MPa, showing a significant improvement compared with single-layer BFRP or CFRP reinforcement. BFRP, as the outer layer, has advantages in resisting initial stress and inhibiting the propagation of microcracks. At the same time, the inner CFRP provides better load-bearing capacity and stiffness due to its higher elastic modulus.

However, the stress–strain curves in Figure 14b reveal different mechanical behaviors when BFRP and CFRP are used as the outer reinforcement layers, respectively. NBC exhibits a relatively early decline in stress after reaching the ultimate stress. It indicates that while the BFRP outer layer offers good ductility under high stress, it struggles to maintain stress growth before instability occurs. BFRP’s stiffness may not fully support the high stress induced by the inner CFRP layer. The performance of the epoxy resin adhesive also plays a critical role in this process. The strength of the bonding interface determines the efficiency of stress transfer between the inner and outer FRP layers [35]. Insufficient bonding can weaken the overall effectiveness of the composite material, making delamination or slippage more likely, especially under high-stress conditions.

For NBG and NBA, despite their relatively lower elastic moduli, the axial compressive strengths reached 48.44 MPa and 45.02 MPa, respectively. This suggests that the presence of the BFRP outer layer partially compensates for the stiffness deficiencies of the inner GFRP and AFRP layers, preventing premature failure under high pressure. Additionally, the stress–strain curves of NBG and NBA indicate that these specimens have weaker stress maintenance capabilities after reaching ultimate stress. However, this also implies that there is potential for improvement in their reinforcement effectiveness under high-strain conditions, which could be a focus of future research and development.

#### 3.3.3. GFRP as the Outer Layer

**Figure 15:**
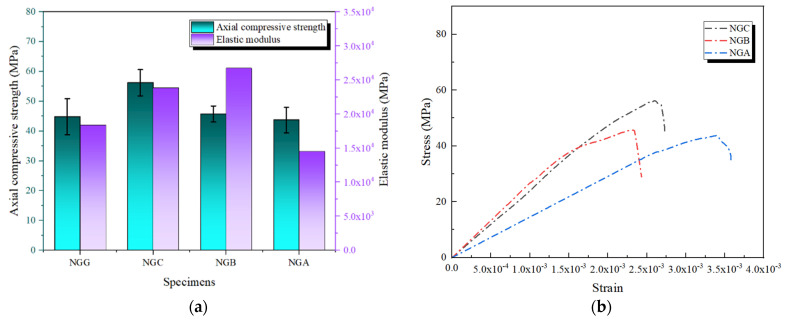
Axial compressive performance of composite reinforcement with GFRP as the outer layer: (**a**) axial compressive strength and elastic modulus; (**b**) stress–strain curves.

Figure 15 presents the experimental results when GFRP is used as the outer reinforcement layer. As shown in Table 5, GFRP exhibits relatively high ductility but has a lower elastic modulus [36]. This characteristic means that its primary contribution to composite reinforcement enhances ductility and promotes uniform stress distribution. The experimental results show that the axial compressive strength of the NGC specimen reached 56.22 MPa. This value is higher than that achieved with single-layer GFRP reinforcement and is notably superior to NGB and NGA. Despite GFRP’s lower stiffness, the high elastic modulus of the inner CFRP layer effectively compensates for the outer layer’s limitations, thereby enhancing the load-bearing capacity of the specimen. The stress–strain curve of NGC displays significant elasticity, with a prolonged strain retention phase after reaching peak stress, indicating good toughness. This suggests that GFRP as the outer layer can effectively disperse stress concentrations, reducing the risk of local failure and, thus, delaying the failure process of the concrete. In contrast, the stress–strain curves of NGB and NGA exhibit stress decline at an earlier stage, indicating that without a high-stiffness inner layer, the GFRP outer layer struggles to maintain a prolonged strain phase under high-stress conditions.

Further analysis of the elastic modulus data reveals that the elastic modulus of the NGC specimen is significantly higher than that of other composite combinations, further validating the critical role of the CFRP inner layer in enhancing overall stiffness. The lower elastic modulus values of NGB and NGA indicate that their inner layer materials (BFRP and AFRP) failed to provide sufficient support in the composite reinforcement. This phenomenon is influenced not only by the intrinsic physical properties of the materials but also by the performance of the epoxy resin adhesive. The bonding strength and toughness of the epoxy resin determine the bond quality between the GFRP outer layer and the inner layer materials [37]. If the adhesive fails to transfer stress or fractures under high stress effectively, the overall performance of the composite material will be compromised.

#### 3.3.4. AFRP as the Outer Layer

**Figure 16:**
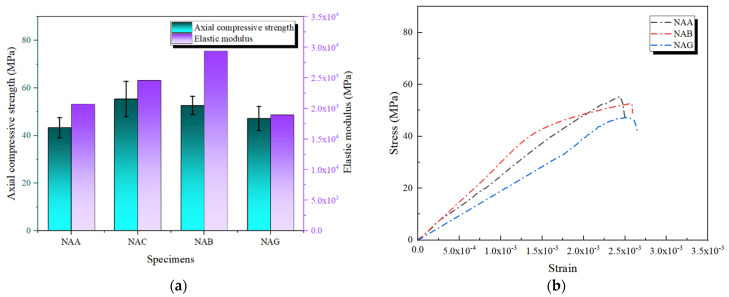
Axial compressive performance of composite reinforcement with AFRP as the outer layer: (**a**) axial compressive strength and elastic modulus; (**b**) stress–strain curves.

AFRP exhibits high toughness and impact resistance but has relatively low tensile elastic modulus and strength. Therefore, its reinforcement effectiveness largely depends on the performance of the inner FRP layer. As shown in Figure 16a, the axial compressive strengths of NAC and NAB are 55.38 MPa and 52.66 MPa, respectively. These values, combined with the stress–strain curves in Figure 16b, demonstrate the impressive load-bearing capacity and prolonged plastic phases of these specimens, reflecting their strong energy absorption capabilities. In contrast, the axial compressive strength of NAG is only 47.19 MPa, falling below 50 MPa, which highlights the limitation of the GFRP inner layer’s stiffness. Additionally, the high toughness of AFRP significantly enhances the structure’s toughness reserve under ultimate conditions, reducing the risk of brittle failure [28].

Furthermore, the analysis of the elastic modulus supports the above conclusions. The elastic modulus of NAC is 2.459 × 10^4^ MPa, while that of the NAB specimen is 2.938 × 10^4^ MPa. This indicates that the CFRP and BFRP inner layers are crucial in increasing overall stiffness. In comparison, the elastic modulus of NAG is only 1.894 × 10^4^ MPa, further reflecting the limitations of the GFRP inner layer in providing sufficient stiffness support. These results suggest that while the AFRP outer layer offers high toughness and ductility, its reinforcement effectiveness heavily depends on the elastic modulus and strength of the inner FRP material. This information is crucial for understanding and predicting the performance of composite materials.

## 4. Finite Element Numerical Simulation

In the Materials and Methods Section, axial compression tests validated the effects of different FRP reinforcement schemes on the mechanical performance of concrete specimens. To further understand the mechanical mechanisms of various reinforcement schemes, this study employed ABAQUS finite element software to perform numerical simulations on four groups of specimens: NDB, NC, NCC, and NCB. By constructing accurate finite element models, it is possible to observe the stress distribution in the FRP layers, deformation patterns, and their impact on the internal stress field of the concrete [38].

### 4.1. Constitutive Relationships

#### 4.1.1. Constitutive Relationship of Concrete

Concrete, as a heterogeneous brittle material, exhibits mechanical behavior influenced by complex stress states. To accurately simulate the mechanical characteristics of concrete under axial compression, the concrete damage plasticity (CDP) model was adopted, as shown in Equations (2) and (3) [39]:

(2) $$\sigma_{c}=E_{0}\varepsilon_{\Delta c}(1-D_{c})$$

(3) $$\sigma_{t}=E_{0}\varepsilon_{\Delta t}(1-D_{t})$$

where *σ_c_*—compressive stress of the concrete;

*σ_t_*—tensile stress of the concrete;

*E*_0_—initial elastic modulus of the concrete;

*ε*_∆*c*_—difference between compressive strain and plastic compressive strain of the concrete;

*ε*_∆*t*_—difference between tensile strain and plastic tensile strain of the concrete;

*D_c_*—compressive damage coefficient of the concrete;

*D_t_*—tensile damage coefficient of the concrete (the damage coefficients range from [0, 1], with higher values indicating more severe damage).

#### 4.1.2. Constitutive Relationship of FRP

In finite element simulations, FRP materials are typically considered linear elastic materials [40] because FRP generally exhibits linear elastic behavior up to the point of failure. Therefore, in this study, the stress–strain relationship of FRP is simplified to a linear relationship, with its mechanical behavior characterized by Young’s modulus and Poisson’s ratio. The Young’s modulus and strength values for different types of FRP were determined based on experimental data. Furthermore, the failure criterion for FRP was based on the maximum strain criterion where the material is considered to fail once the strain reaches a specific critical value [41]. The maximum strain criterion assumes that the material will experience fracture failure when the FRP strain reaches a defined threshold [42,43]. This approach not only simplifies the failure simulation process for FRP-reinforced structures but also effectively captures the stress concentration and fracture behavior of FRP under compression. Based on experimental data, strain limit values were established for different types of FRP (CFRP, BFRP, GFRP, and AFRP) to ensure that the simulation accurately reflects the true mechanical performance of the materials. The use of this criterion allows the model to maintain computational efficiency while adequately describing the failure behavior of both the FRP and concrete.

### 4.2. Parameter Settings

#### 4.2.1. Element Selection and Mesh Generation

**Figure 17:**
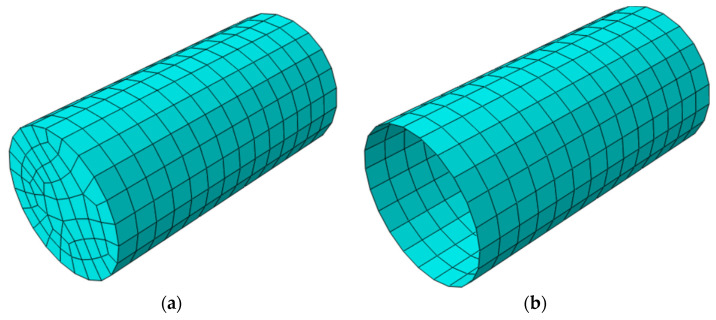
ABAQUS mesh generation: (**a**) concrete; (**b**) FRP.

In finite element simulations, the appropriate selection of element types and mesh generation is crucial for ensuring model accuracy and computational efficiency [44]. To ensure a balance between simulation accuracy and computational efficiency, several models with varying element sizes were tested to determine the optimal element size. Ultimately, the selected element size was similar to that used by Ji et al. [45,46]. This element size was chosen to accurately capture the structural behavior without causing excessive computational load or model divergence. Additionally, to maintain model precision and ensure convergence, 1375 C3D8R solid elements (8-node linear reduced integration hexahedral elements) were employed for the concrete portion. These elements are well suited for simulating the three-dimensional stress states of concrete, effectively capturing complex stress distributions and deformation characteristics. For the FRP materials, 472 S4R shell elements (4-node linear reduced integration shell elements) were utilized, given that the thickness of FRP is significantly smaller than other dimensions, and the analysis primarily focuses on in-plane strength [47]. This type of element accurately simulates the in-plane stress distribution of FRP while reducing computational effort and improving efficiency. The mesh generation for concrete and FRP is illustrated in Figure 17.

#### 4.2.2. Boundary Conditions and Loading

**Figure 18:**
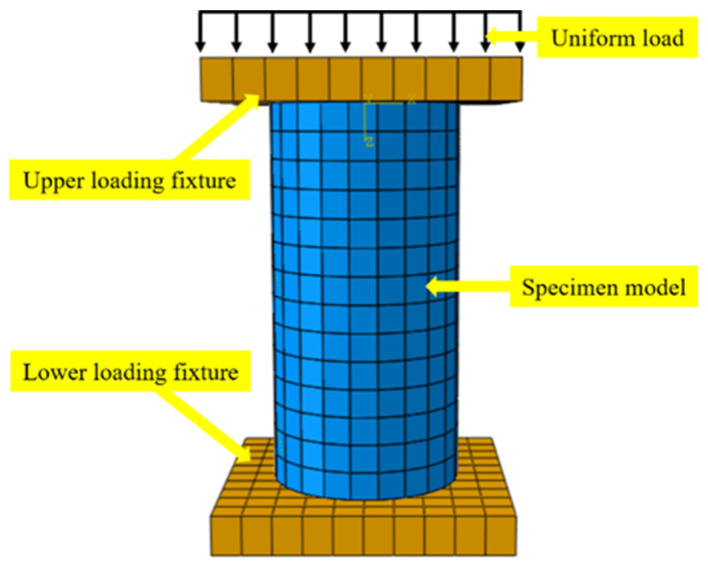
Schematic diagram of the ABAQUS model.

As shown in Figure 18, the ABAQUS model schematic illustrates the loading scheme for the finite element simulation. The model uses a static general analysis type [46] to simulate the stress response of the concrete specimen under axial compression loads. The initial increment size is set to 0.01 [45,48,49], with the maximum and minimum increments set to 1 × 10^−5^ and 0.1, respectively, while the geometric nonlinearity option is deactivated. In the field output manager, the output frequency is set to “every x time units” to capture key moments of the analysis. To ensure that the ABAQUS model aligns with experimental conditions, the mechanical loading position and method are carefully defined. Steel fixtures are created on the top and bottom sides of the concrete model to apply the axial compression load. The six degrees of freedom for the bottom fixture, including X, Y, Z, RXY, RYZ, and RZX, are fully constrained. A discretization method is used to establish the contact between the concrete and the steel fixtures, with the contact type set to “Surface-to-surface contact (Standard).” The parameters for tangential and normal behavior in the “Edit Interaction” settings are also defined. The contact between the concrete and the fixtures is configured as “hard contact” with “no friction.” An evenly distributed load is applied to the concrete model through the upper steel fixture, corresponding to a stress rate of 0.05 MPa/s. The simulation is terminated when the CFRP material reaches its ultimate strain, indicating that the model has experienced full failure.

### 4.3. Finite Element Results Analysis

**Figure 19:**
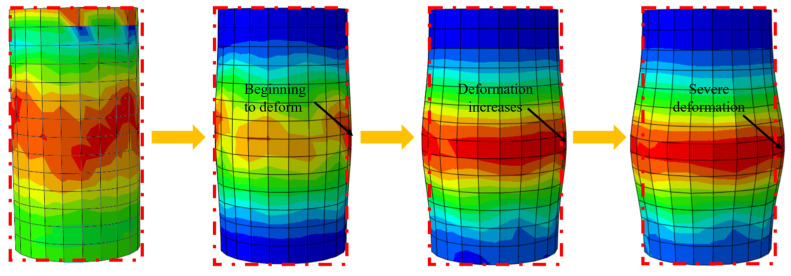
Deformation of the specimen during axial compression.

Taking the NCB specimen with a diameter of 100 mm and a height of 200 mm as an example, Figure 19 illustrates the deformation of the FRP-wrapped concrete model during axial compression. To make the deformation process more visually apparent, the deformation scale factor is magnified 30 times. It can be observed that during the axial compression process, the deformation of the specimen is primarily concentrated in the middle region. As the axial load increases, the degree of deformation intensifies progressively, transitioning from slight deformation at the beginning to severe deformation at failure, exhibiting significant stress concentration. This corresponds to the gradual failure of the FRP confinement around the concrete. At the initial loading stage, the FRP effectively restrains the deformation of the concrete. However, as the pressure increases, the FRP’s confinement capacity gradually diminishes, leading to the most pronounced expansion in the middle region. The stress and strain in the middle of the specimen progressively accumulate until the FRP reaches its ultimate strength and strain, ultimately resulting in fracture failure.

**Figure 20:**
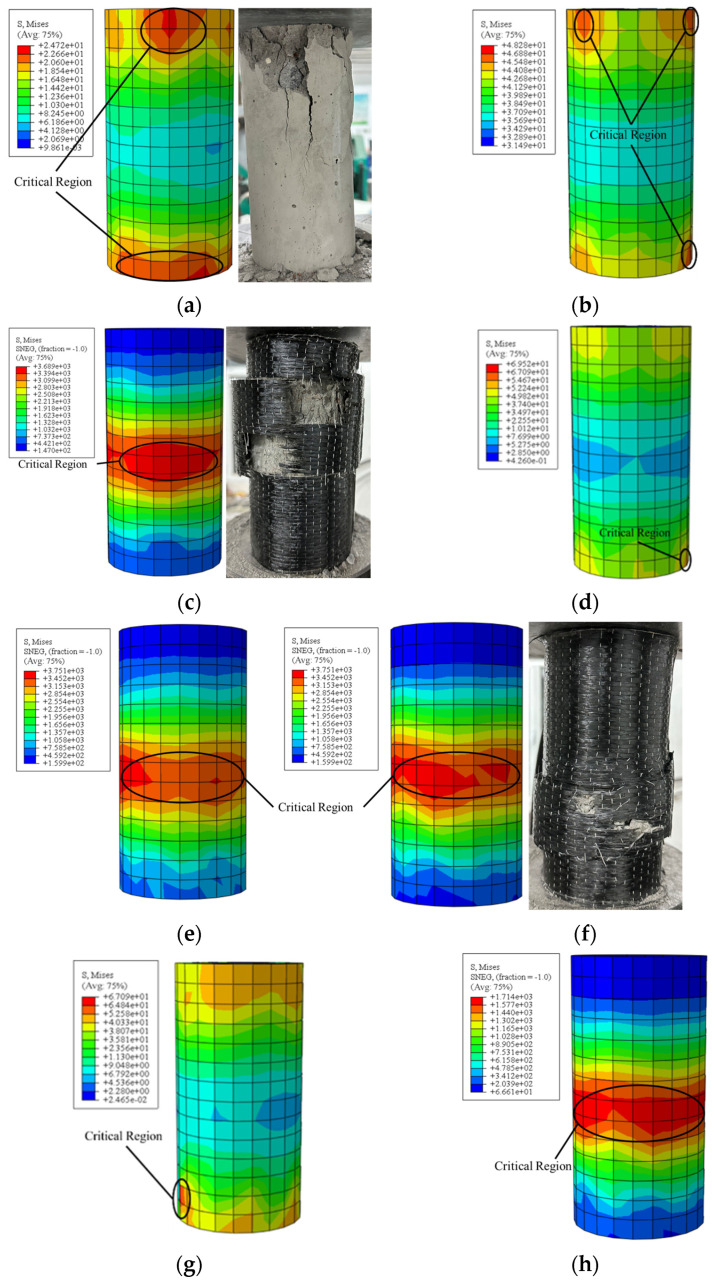

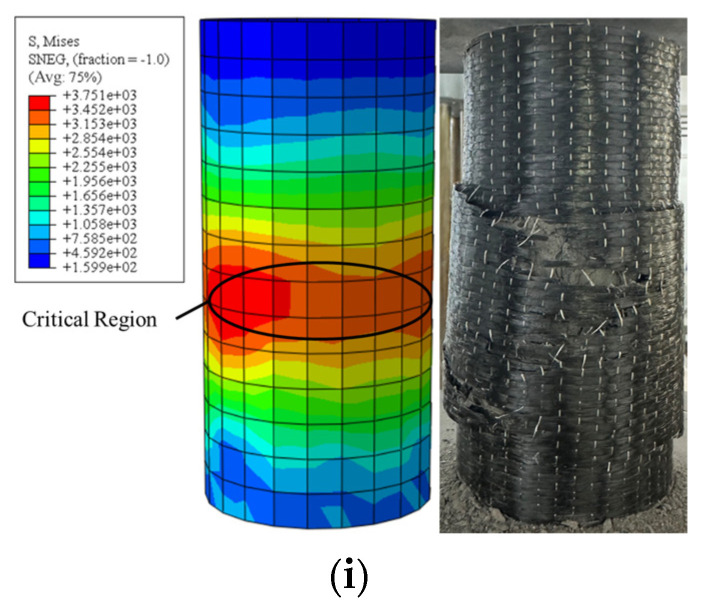
Stress contour plot and experimental image: (**a**) NDB; (**b**) NC-concrete; (**c**) NC-CFRP; (**d**) NCC-concrete; (**e**) NCC-CFRP-inside; (**f**) NCC-CFRP-outside; (**g**) NCB-concrete; (**h**) NCB-BFRP; (**i**) NCB-CFRP.

The simulation results for NDB, NC, NCC, and NCB are shown in Figure 20. These stress contour plots visually depict the internal stress distribution, load-bearing characteristics, and critical regions within the concrete specimens during loading. The stress contour of NDB reveals significant stress concentration, particularly in the loading surface and central regions. Due to the absence of external reinforcement, the concrete is more susceptible to localized stress concentration under compression. In contrast, the stress distribution in the NC concrete model is more uniform, especially along the surface and edge regions. This indicates that the high elastic modulus of CFRP effectively enhances the overall stiffness of the concrete, mitigating stress concentration to some extent. However, localized stress concentration still occurs near the loading area, representing the concrete’s most critical region during the loading process. In the NCC model, the double-layer CFRP reinforcement further equalizes the stress distribution in the concrete. This suggests that double-layer CFRP reinforcement significantly improves the specimen’s overall stiffness, reduces stress concentration, and enhances the structural load-bearing capacity [50]. Comparatively, although the composite-reinforced NCB exhibits relatively uniform stress distribution on the surface and internal regions, the lower stiffness of the BFRP inner layer leads to some degree of stress concentration in the loading area. This indicates that while NCB performs better in stress distribution than the single-layer CFRP reinforcement scheme, its reinforcement effectiveness is not as pronounced as that of the double-layer CFRP. Whether in double-layer or composite reinforcement, the critical regions in the concrete are significantly smaller than those in the non-FRP reinforced and single-layer reinforced groups.

By comparing the stress distribution contour plots of the CFRP and BFRP reinforcement layers, it can be observed that the stress is primarily concentrated in the central region of the specimens. This phenomenon is closely related to the deformation behavior of the concrete under compression [51]. As the concrete is compressed, the central region of the specimen typically undergoes greater deformation, resulting in higher stress concentration in that area. Therefore, the central position of the FRP reinforcement layer is often where the critical region is located. This observation aligns with experimental results where FRP reinforcement layers often begin to crack in the central region.

Table 6 presents a comparison between the experimental and finite element simulation results for compressive strength and ultimate compressive strain of different specimens. It can be observed that, although there is some discrepancy between the experimental and simulation results, the simulation generally reflects the mechanical behavior observed in the tests. Specifically, the error between the simulated and measured compressive strength values is within 12%, and the relative error for ultimate compressive strain is below 14%, indicating a high level of accuracy in the simulation results. The primary reasons for the discrepancies may be attributed to the interface effects between FRP layers, variations in the properties of FRP materials, and the simplified treatment of stress concentration regions during the loading process.

## 5. Conclusions

This study investigated the effects of different types of FRP and their composite configurations on the mechanical performance of concrete specimens under axial compression. Through single-layer, double-layer, and composite FRP reinforcement tests, as well as ABAQUS finite element simulations, the following conclusions were drawn:

(1) Among the single-layer reinforcements, CFRP exhibited the best performance. This is attributed to its high tensile strength and elastic modulus, which effectively constrained concrete deformation, thereby enhancing the load-bearing capacity of the specimens. BFRP, GFRP, and AFRP demonstrated better ductility and energy absorption, but their reinforcement effects under high-stress conditions were relatively limited.
(2) Double-layer CFRP reinforcement showed the most optimal overall performance, significantly improving the compressive strength and stiffness of the specimens. In the ABAQUS simulations, the NCC specimens exhibited more uniform stress distribution with fewer instances of localized stress concentration. While other double-layer combinations, such as those involving BFRP and GFRP, also showed some reinforcement effects, their contributions to overall structural stiffness were limited due to their lower elastic modulus.
(3) In composite reinforcement when CFRP was used as the outer layer, it formed a solid synergistic effect with the inner FRP. This was particularly evident when combined with BFRP or GFRP, resulting in high axial compressive strength and stiffness. However, when BFRP and GFRP were used as the outer layers, although there was an improvement in ductility, the enhancement in axial compressive performance and stiffness was not as pronounced as with CFRP. When AFRP was used as the outer reinforcement material, its high toughness somewhat enhanced the structure’s ductility. However, its overall reinforcement effectiveness was mainly dependent on the performance of the inner FRP, resulting in a relatively weaker performance overall.

## Figures and Tables

**Table 1:** Basic physical properties of the four types of FRP.

FRP Type	Thickness (mm)	Tensile Strength (MPa)	Tensile Elastic Modulus (MPa)	Elongation (%)
CFRP	0.167	3520	267	1.78
BFRP	0.190	3000	120	1.60
GFRP	0.200	2500	80	2.30
AFRP	0.155	2106	117.8	1.75

**Table 2:** Physical and chemical properties of cement.

Test Items	Fineness/%	Setting Time/(min)		Compressive Strength		Flexural Strength	
		Initial Setting Time	Final Setting Time	3 d	28 d	3 d	28 d
Standard value	≤10.0	≥45	≤600	≥17	≥42.5	≥3.5	≥6.5
Detection value	3.7	148	374	25.7	48.2	4.7	7.5

**Table 3:** Concrete mix proportions.

Materials	Cement	Coarse Aggregate	Fine Aggregate	Water
Content (kg/m^3^)	213	387	635	1169

**Table 4:** FRP Bonding Design Scheme.

Specimen	Number of FRP Layers	Outer FRP Type	Inner FRP Type
BDB	0	—	—
NC	1	CFRP	—
NB	1	BFRP	—
NG	1	GFRP	—
NA	1	AFRP	—
NCC	2	CFRP	CFRP
NBB	2	BFRP	BFRP
NGG	2	GFRP	GFRP
NAA	2	AFRP	AFRP
NCB	2	CFRP	BFRP
NCG	2	CFRP	GFRP
NCA	2	CFRP	AFRP
NBC	2	BFRP	CFRP
NBG	2	BFRP	GFRP
NBA	2	BFRP	AFRP
NGC	2	GFRP	CFRP
NGB	2	GFRP	BFRP
NGA	2	GFRP	AFRP
NAC	2	AFRP	CFRP
NAB	2	AFRP	BFRP
NAG	2	AFRP	GFRP

**Table 5:** Axial compressive performance test results of different specimens.

Specimen	Axial Compressive Strength (MPa)	Strength Improvement Over Non-FRP Group (%)	Ultimate Load (MPa)	Ultimate Compressive Strain (×10^−3^)	Elastic Modulus (×10^4^ MPa)
NDB	24.66	0.00	28.52	1.29	1.969
NC	45.07	82.77	46.59	1.56	3.275
NB	39.64	60.75	42.14	1.97	2.063
NG	36.01	46.03	42.53	1.72	2.015
NA	34.07	38.16	37.49	2.19	1.712
NCC	77.81	215.53	78.03	1.89	4.453
NBB	55.26	124.09	58.19	2.51	2.383
NGG	44.85	81.87	47.64	2.21	1.840
NAA	43.34	75.75	44.92	3.35	2.070
NCB	72.72	194.89	73.25	2.55	3.201
NCG	58.87	138.73	58.93	2.26	3.248
NCA	48.98	98.62	50.74	2.50	2.090
NBC	64.59	161.92	66.85	1.43	5.396
NBG	48.44	96.43	49.87	1.92	2.262
NBA	45.02	82.56	47.19	2.66	1.803
NGC	56.22	127.98	57.71	2.60	2.384
NGB	45.73	85.44	46.08	2.30	2.672
NGA	43.74	77.37	46.19	3.39	1.453
NAC	55.38	124.57	56.36	2.44	2.459
NAB	52.66	113.54	53.73	2.57	2.938
NAG	47.19	91.36	49.65	2.54	1.894

**Table 6:** Measured and Simulated Results.

Specimen	Axial Compressive Strength (MPa)		Relative Error (%)	Ultimate Compressive Strain (×10^−3^)		Relative Error (%)
	Measured Results	Simulated Results		Measured Results	Simulated Results	
NDB	24.66	22.58	8.43	1.29	1.18	8.52
NC	45.07	43.74	2.95	1.56	1.35	13.46
NCC	77.81	68.52	11.94	1.89	1.74	7.94
NCB	72.72	64.19	11.73	2.55	2.27	10.98

## Data Availability

The data presented in this study are available on request from the corresponding author. The data are not publicly available due to privacy.

## References

[1] Cao X.-Y., Shen D., Feng D.-C., Wang C.-L., Qu Z., Wu G. (2022). Seismic retrofitting of existing frame buildings through externally attached sub-structures: State of the art review and future perspectives. J. Build. Eng..

[2] Hu J., Hu J., Zhang S., Zhang S., Chen E., Chen E., Li W., Li W. (2022). A review on corrosion detection and protection of existing reinforced concrete (RC) structures. Constr. Build. Mater..

[3] Mo J., Uy B., Li D., Thai H.-T., Tran H. (2021). A review of the behaviour and design of steel–concrete composite shear walls. Structures.

[4] Niu J., Xu W., Li J., Liang J. (2021). Influence of Cross-Sectional Shape on the Mechanical Properties of Concrete Canvas and CFRP-Reinforced Columns. Adv. Mater. Sci. Eng..

[5] Dai J., Bai Y., Teng G.J. (2011). Behavior and Modeling of Concrete Confined with FRP Composites of Large Deformability. J. Compos. Constr..

[6] Liu X., Wu T., Chen H., Liu Y. (2020). Compressive stress-strain behavior of CFRP-confined lightweight aggregate concrete reinforced with hybrid fibers. Compos. Struct..

[7] Hemida OA R., Abdalla H.A., Fouad HE E. (2023). Flexural behaviour of recycled reinforced concrete beams strengthened/repaired with CFRP laminates. J. Eng. Appl. Sci..

[8] Ouyang L.J., Gao W.Y., Zhen B., Lu Z.D. (2017). Seismic retrofit of square reinforced concrete columns using basalt and carbon fiber-reinforced polymer sheets: A comparative study. Compos. Struct..

[9] Khalifa A., Nanni A. (2000). Improving shear capacity of existing RC T-section beams using CFRP composites. Cem. Concr. Compos..

[10] Raza A., Ali B., Nawaz M.A., Ahmed I. (2020). Structural performance of FRP-RC compression members wrapped with FRP composites. Structures.

[11] (2012). Technical Code for Safety Appraisal of Engineering Structural Strengthening Materials.

[12] Mazzuca P., Pisani B., Firmo J.P., Ombres L. (2024). Tensile and bond properties at elevated temperatures of a PBO-FRCM composite system for strengthening concrete elements: Experimental and analytical investigations. Constr. Build. Mater..

[13] (2011). Pebbie and Crushed Stone for Building.

[14] (2006). Standard for Technical Requirements and Test Method of Sand and Crushed Stone (or Gravel) for Ordinary Concrete.

[15] (2007). Common Portland Cement.

[16] (2006). Standard if Water Fir Cibcreke.

[17] (2002). Standard for Test Method of Performance on Ordinary Fresh Concrete.

[18] (2019). Standard for Test Methods of Concrete Physical and Mechanical Properties.

[19] Raza A., Khan Q.Z., Ahmad A. (2020). Prediction of axial compressive strength for FRP-confined concrete compression members. KSCE J. Civ. Eng..

[20] Ahmet Yaşar B., Kose M.M., Avğın S., Temiz H. (2020). Determination of elasticity modulus for low strength concrete. El-Cezeri.

[21] Benmokrane B., Zhang B., Chennouf A. (2000). Tensile properties and pullout behaviour of AFRP and CFRP rods for grouted anchor applications. Constr. Build. Mater..

[22] Ou Y., Zhu D., Zhang H., Huang L., Yao Y., Li G., Mobasher B. (2016). Mechanical characterization of the tensile properties of glass fiber and its reinforced polymer (GFRP) composite under varying strain rates and temperatures. Polymers.

[23] Qaidi S., Al-Kamaki Y.S.S., Al-Mahaidi R., Mohammed A.S., Ahmed H.U., Zaid O., Althoey F., Ahmad J., Isleem H.F., Bennetts I. (2022). Investigation of the effectiveness of CFRP strengthening of concrete made with recycled waste PET fine plastic aggregate. PLoS ONE.

[24] Tu J., Xie H., Gao K., Li Z., Zhang J. (2019). Durability prediction of GFRP rebar based on elastic modulus degradation. Front. Mater..

[25] Li Y., Wang Y., Ou J. (2014). Mechanical behavior of BFRP-steel composite plate under axial tension. Polymers.

[26] Kim Y.J. (2010). Flexural response of concrete beams prestressed with AFRP tendons: Numerical investigation. J. Compos. Constr..

[27] Yu Q.-Q., Zhao Y.-Z., Wu H., Wang Z., Zhu Z.-Y. (2023). Experimental study on AFRP-to-concrete bond behavior subjected to underground water. Compos. Struct..

[28] Anas S.M., Shariq M., Alam M. (2022). Performance of axially loaded square RC columns with single/double confinement layer (s) and strengthened with C-FRP wrapping under close-in blast. Mater. Today Proc..

[29] Yuan C., Chen W., Pham T.M., Hao H., Chen L., Zhang M. (2020). New epoxy anchor for better bonding between FRP sheets and concrete. Constr. Build. Mater..

[30] Durgadevi S., Karthikeyan S., Lavanya N., Kavitha C. (2021). A review on retrofitting of reinforced concrete elements using FRP. Mater. Today Proc..

[31] Ma G., Chen X., Yan L., Hwang H.-J. (2020). Effect of pre-damage on compressive properties of circular RC columns repaired with BFRP composites: Testing and modeling. Compos. Struct..

[32] Ranolia K.V., Thakkar B.K., Rathod J.D. (2013). Effect of different patterns and cracking in FRP wrapping on compressive strength of confined concrete. Procedia Eng..

[33] Jiang F., Han X., Wang Y., Wang P., Zhao T., Zhang K. (2022). Effect of freeze-thaw cycles on tensile properties of CFRP, bond behavior of CFRP-concrete, and flexural performance of CFRP-strengthened concrete beams. Cold Reg. Sci. Technol..

[34] Tang Y., Sun Z., Wei Y., Zou X. (2022). Compressive behavior and design method of BFRP bars constrained with a BFRP spiral with different spacings in concrete members. Eng. Struct..

[35] Li P.-D., Zhao Y., Wu Y.-F., Lin J.-P. (2023). Effect of defects in adhesive layer on the interfacial bond behaviors of externally bonded CFRP-to-concrete joints. Eng. Struct..

[36] Liu Y., Zhang H.-T., Tafsirojjaman T., Dogar A.U.R., AlAjarmeh O., Yue Q.-R., Manalo A. (2022). A novel technique to improve the compressive strength and ductility of glass fiber reinforced polymer (GFRP) composite bars. Constr. Build. Mater..

[37] Yang S., Liu C., Sun Z., Xu M., Feng Y. (2022). Effects of resin pre-coating treatment and fibre reinforcement on adhesive bonding between CFRP sheet and concrete. Compos. Struct..

[38] Hardan S.A., Aules W.A. (2022). Analysis of CFRP confined concrete cylinders by using ABAQUS software. Tikrit J. Eng. Sci..

[39] Le Thanh C., Minh H.L., Sang-To T. (2022). A nonlinear concrete damaged plasticity model for simulation reinforced concrete structures using ABAQUS. Frat. Ed Integrità Strutt..

[40] Naser M.Z., Hawileh R.A., Abdalla J. (2021). Modeling strategies of finite element simulation of reinforced concrete beams strengthened with FRP: A review. J. Compos. Sci..

[41] Zheng Z., Du Y., Chen Z., Li S., Niu J. (2021). Experimental and theoretical studies of FRP-Steel composite plate under static tensile loading. Constr. Build. Mater..

[42] Lam L., Teng J.G. (2003). Design-oriented stress–strain model for FRP-confined concrete. Constr. Build. Mater..

[43] Kwan A.K.H., Dong C.X., Ho J.C.M. (2015). Axial and lateral stress–strain model for FRP confined concrete. Eng. Struct..

[44] Kudela J., Matousek R. (2022). Recent advances and applications of surrogate models for finite element method computations: A review. Soft Comput..

[45] Ji Y., Liu W., Jia Y., Li W. (2021). Durability investigation of carbon fiber reinforced concrete under salt-freeze coupling effect. Materials.

[46] Ji Y., Li Z., Xu W., Li W. (2024). Mechanical damage mechanism investigation on CFRP strengthened recycled red brick concrete. Rev. Adv. Mater. Sci..

[47] Raza A., Shah S.A.R., Khan A.R., Aslam M.A., Khan T.A., Arshad K., Hussan S., Sultan A., Shahzadi G., Waseem M. (2020). Sustainable FRP-confined symmetric concrete structures: An application experimental and numerical validation process for reference data. Appl. Sci..

[48] Ji Y., Pei Z., Xu W., Li Z., Li Y., Jia Y. (2024). Deterioration performance analysis of recycled brick concrete subjected to freezing and thawing effect. Case Stud. Constr. Mater..

[49] Ji Y., Wang D. (2022). Durability of recycled aggregate concrete in cold regions. Case Stud. Constr. Mater..

[50] Raza A., Rafique U., Masood B., Ali B., Haq F.U., Nawaz M.A. (2021). Performance evaluation of hybrid fiber reinforced low strength concrete cylinders confined with CFRP wraps. Structures.

[51] Wang H.C., Zhao J., Li J., Liu K., Braithwaite C.H., Zhang Q.B. (2021). Dynamic mechanical properties and fracturing behaviour of concrete under biaxial compression. Constr. Build. Mater..

